# Locking plate versus K-wires and cast fixation in lateral closing-wedge osteotomy for cubitus varus deformity

**DOI:** 10.3389/fped.2024.1344283

**Published:** 2024-02-09

**Authors:** Jianghua Liu, Youzhi He, Qiang Shi, Yongfu Wang

**Affiliations:** ^1^ Department of Orthopaedics, The First Affiliated Hospital, Hengyang Medical School, University of South China, Hengyang, China; ^2^ Department of Spine Surgery, The Affiliated Changsha Central Hospital, Hengyang Medical School, University of South China, Changsha, China

**Keywords:** cubitus varus, lateral closing-wedge osteotomy, K-wire, locking plate, osteotomy

## Abstract

**Background:**

The aim of this study was to assess the clinical and radiographic outcomes of cubitus varus treatments based on different fixation methods: Locking plate vs. Kirschner-wires (K-wires) and cast fixation.

**Methods:**

This retrospective study of 28 patients was performed in lateral-wedge osteotomy for cubitus varus deformity in our hospital from July 2018 to July 2020. 14 patients in group A were treated by locking plate after lateral closing-wedge osteotomy, whereas other 14 patients were treated by K-wires in group B. We measured the bony union and carrying angle. The clinical and radiographic outcomes were assessed according to the Bellemore criteria.

**Results:**

No nonunion, neurovascular injury or myositis ossificans was noted at follow-up. In group A, 1 patient with lateral condylar prominence was found. In group B, 2 patients with pinning site infection were treated successfully with oral antibiotics and 2 patients needed revision surgery for residual varus. According to the Bellemore criteria, statistically significant difference was noted between the two groups *(P = 0.0458)*. In the present study, no statistically significant difference was noted in the length of incision and operation time between the 2 groups *(P *>* 0.05)*. However, the postoperative carrying angle was significantly different at final follow-up between the 2 groups *(P < 0.01)*.

**Conclusions:**

Compared with K-wires and cast fixation, we recommend the wedge osteotomy with lateral locking plate to treat the cubitus varus deformity because locking plate could achieve better functional and cosmetic results and stabilize the distal humerus rigidly.

## Background

Supracondylar humerus fractures are common in children, while the incidence of cubitus varus following this type of fracture is between 7% and 40% (1–3). This deformity belongs to a three-dimensional (3D) deformity, which includes medial tilting in the coronal plane, extension in the sagittal plane, and internal rotation of the distal fragment in the horizontal plane (4, 5). In the majority of cases, poor reduction after displaced supracondylar humerus fractures may lead to instability, fragment tilt and subsequent varus of the elbow joint, thus increase the incidence of cubitus varus deformity (6). Meanwhile, remodeling of this deformity does not improve with age. Therefore, the child's parents often request corrective surgery to improve the appearance or functional problems of the elbow (3, 7, 8). Surgical corrections for moderate-to-severe deformities have been introduced to improve cosmetic conditions or prevent functional impairment such as restricted range of motion, instability, and ulnar nerve neuropathy (9). Various methods for correction of cubitus varus deformity have been emphasized to correct 3D deformity and lateral closing-wedge osteotomy is always performed by most orthopedic surgeons, which demonstrates more effective than dome osteotomy or valgus osteotomy (10, 11).

Recently, Li et al. indicated that lateral external fixation had a limited advantage over single-plate internal fixation for treatment of cubitus varus (12). However, no studies compared the clinical and radiologic outcomes of locking plate against Kirschner-wires (K-wires) and cast fixation. This study aims to compare the clinical outcomes of patients operated with the locking plate vs. K-wires and cast fixation for cubitus varus deformity.

## Materials and methods

### Patients

This retrospective study of 28 patients was performed to compare locking plate vs. K-wires and cast fixation in lateral-wedge osteotomy for cubitus varus deformity in our hospital from July 2018 to July 2020. 14 patients in group A were treated by locking plate after lateral closing-wedge osteotomy, whereas other 14 patients were treated by K-wires in group B. There was no significant difference between 2 groups in terms of age, gender, side, carrying angle, and motions in Table 1. The inclusion criteria were (1) malunion of supracondylar humerus fracture; (2) deformity progression, cosmetic unacceptability, functional limitation or long-term sequelae concerns by parents; (3) between 6 and 14 years; (4) underwent lateral closing-wedge osteotomy. The exclusion criteria were (1) regular follow-up for <2 year; (2) lack of imaging data; (3) varus angle was <15°; (4) associated injuries including nerve or other injuries. One single senior surgeon performed all the lateral-wedge osteotomies in our department.

**Table 1:** Comparison of demographic data and characteristics between two groups.

Characteristics	Group A (*n* = 16)	Group B (*n* = 16)	*P* value
Mean age (range), years	9.6 ± 2.1 (6–14)	9.4 ± 2.2 (6–14)	0.727
Gender, *n* (%)			0.723
Male	8 (50.0)	7 (43.8)	
Female	8 (50.0)	9 (56.2)	
Side, *n* (%)			0.719
Left	7 (43.8)	6 (37.5)	
Right	9 (56.2)	10 (62.5)	
Preoperative carrying angle (°)			
Affected side	−20.0 ± 5.8	−21.1 ± 5.6	0.319
Normal side	6.1 ± 1.7	6.8 ± 1.5	0.202
Hospital stay (range), days	8.0 ± 1.8	6.4 ± 1.2	0.003

Patients' functional outcomes were assessed according to the Bellemore criteria at last follow-up (13). This study was approved by the institutional review board of the Affiliated Changsha Central Hospital, Hengyang Medical School. All parents gave written informed consent before participating in this study. All parents gave their written informed consent for the publication of children images.

### Treatment procedure

In both 2 groups, preoperative anteroposterior and lateral radiographs of both upper extremities were evaluated to measure the carrying angle (defined by the longitudinal humeral axis and a line passing through the proximal and distal midpoints of the radius and ulna) and the angle of wedge osteotomy (the affected varus angle plus the carrying angle of the contralateral normal elbow). Firstly, a 3 to 4 cm lateral approach was performed along the distal humerus over the supracondylar ridge to avoid damaging the radial nerve under a tourniquet. Then based on the preoperative measurement, the distal osteotomy line was made parallelly to the elbow joint line just 1–2 cm above the olecranon fossa and the lateral closing-wedge osteotomy was performed.

#### Locking plate fixation (group A)

**Figure 1:**
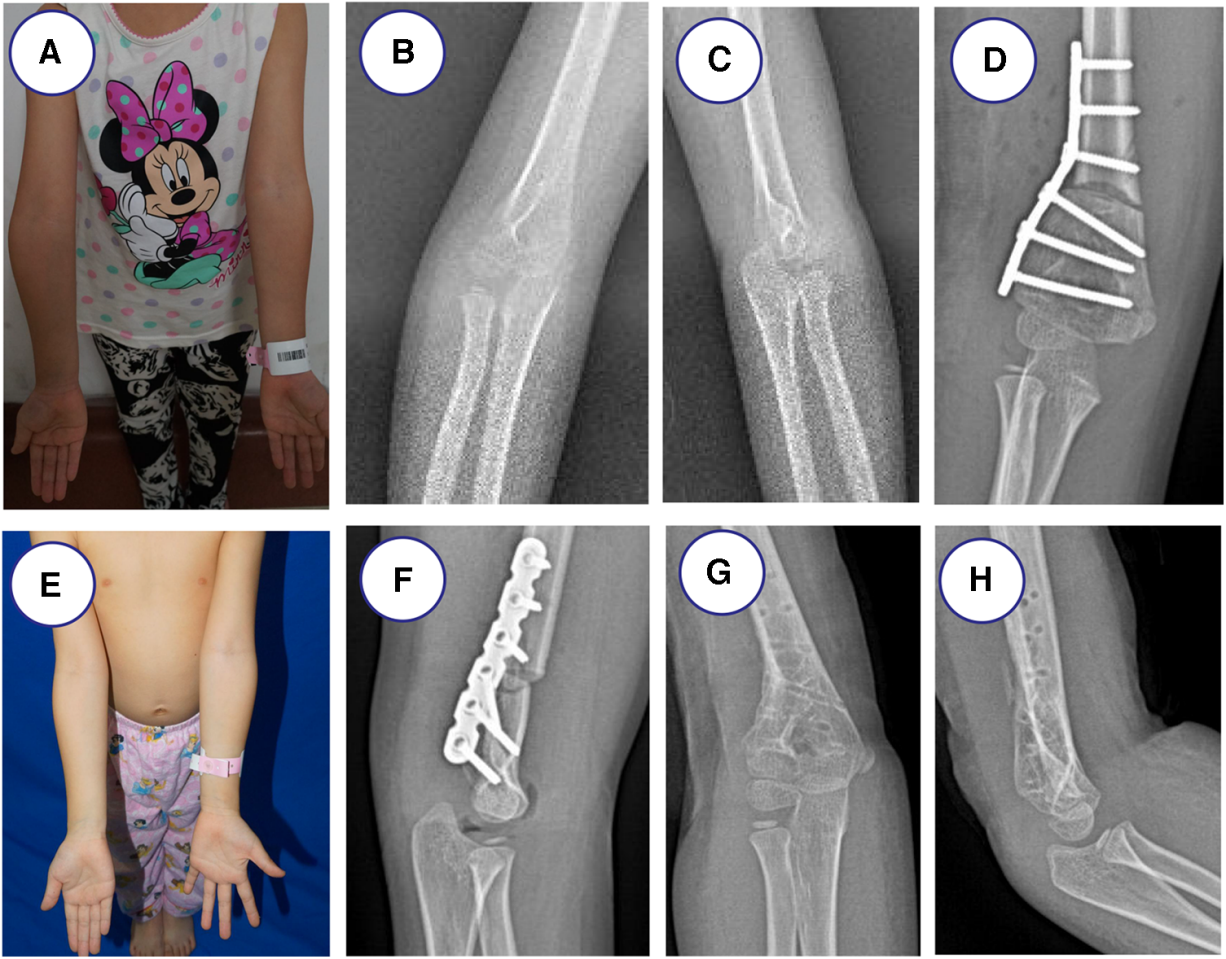
Locking plate fixation of a 9-year-old girl with right cubitus varus owing to right supracondylar humeral fracture. (**A**) Clinical photograph before surgery. (**B,C**) Preoperative radiographs showed right cubitus varus before osteotomy. (**E**) Clinical photograph at the last follow-up time. (**D,F**) Postoperative radiographs show locking plate fixation after lateral closing-wedge osteotomy. (**G,H**) Locking plate fixation was removed after 7 months.

After performing the osteotomy and removing the osteotomy fragment, the pre-bent locking plate was used, then the osteotomy site was fixed with a 4-hole 3.5-mm locking plate and 2 screws on the proximal and distal side of the osteotomy. The screws were inserted via both cortices of distal humerus. The C-arm radiograph was made before closure for final assessment (Figure 1).

#### K-wires and cast fixation (group B)

**Figure 2:**
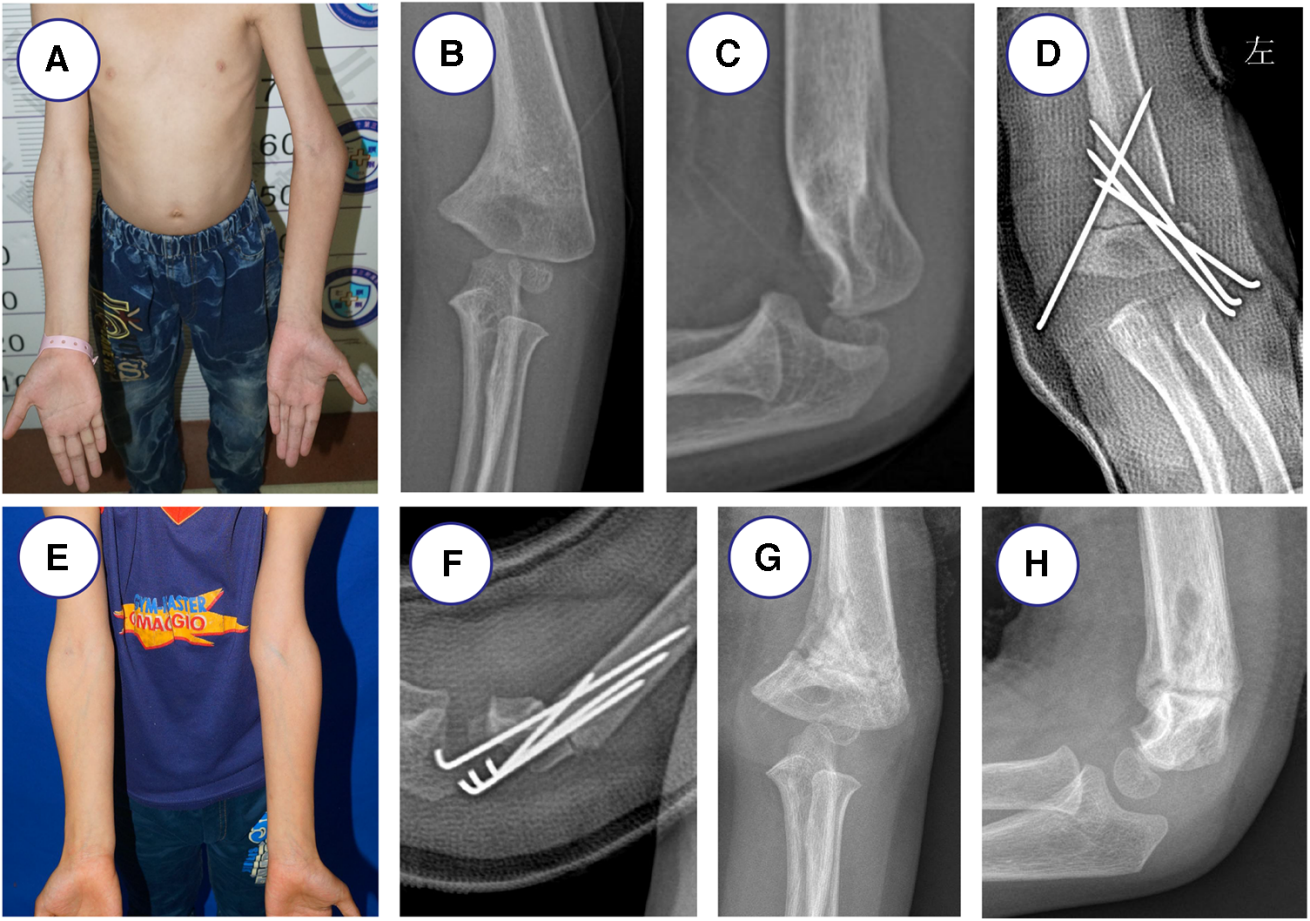
7-year-old boy with left cubitus varus treated with K-wires and cast fixation owing to left supracondylar humeral fracture. (**A**) Clinical photograph before surgery. (**B,C**) Radiographs showed left cubitus varus before surgery. (**E**) Clinical photograph after surgery at the last follow-up time. (**D,F**) Radiographs show K-wires and cast fixation after lateral closing-wedge osteotomy. (**G,H**) K-wires fixation were removed after 2 months.

After a satisfactory carrying angle of the elbow was achieved, one medial K-wire and three lateral cross K-wires (1.5–2.0 mm in diameter) were passed from distal to proximal. The C-arm radiograph was obtained before closure for final assessment to confirm the correct placement of the K-wires during surgery. Then the elbow was immobilized in an above-elbow cast with 90° of elbow flexion. The typical operative stages are shown in Figure 2.

### Follow-up and postoperative management

The mean follow-up was 4.2 years (3 to 5 years) and patients were reviewed at two, four and six months and then every year until skeletal maturity. In Group A, radiographs of the humeral joint were taken regularly until the region of osteotomy completely healed and then the locking plate was removed (at month 6 to 12 postoperatively). In Group B, the K-wires were removed after radiological signs of union (mean 3 months) and changed to elbow brace for another 4 weeks. Clinical evaluations included passive range of elbow joint, the carrying angle, and surgical complications. Patients' functional outcomes were assessed according to the Bellemore criteria at last follow-up.

### Statistical analysis

Quantitative data in this study were statistically analyzed by the SPSS 25.0 software (SPSS, Inc., Chicago, USA) and manifested as count (percentage) or mean ± standard deviation (SD). Student's *t* test, chi-squared test, and Fisher's exact test were applied to analyze the data in this study. Different parameters measured between two groups were evaluated with independent *t* test for continuous variables, and chi-square test or Fisher's exact test for the categorical variables. A *P* value < 0.05 was considered statistically significant.

## Results

No nonunion, neurovascular injury or myositis ossificans was noted at follow-up. No significant difference was found in gender, age, union time, and follow-up period. Adequate healing is usually achieved at three months follow-up time in both groups. At this time the K-wires can be removed in the outpatient clinic in group B. In group A, 1 patient with lateral condylar prominence was found. In group B, 2 patients with pinning site infection were treated successfully with oral antibiotics and 2 patients needed revision surgery (performing lateral closing-wedge osteotomy with locking plate 1 year later) for residual varus.

According to the Bellemore criteria, the functional outcomes of both groups were showed in Table 2. Excellent and good results were considered satisfactory in our study. Statistically significant difference was noted between the two groups *(P = 0.0458)*. In the present study, no statistically significant difference was noted in the length of incision and operation time between the 2 groups *(P *>* 0.05)*. However, the postoperative carrying angle was significantly different at final follow-up between the 2 groups *(P < 0.01)*.

**Table 2:** Comparison of operation data and functional outcomes between two groups.

	Group A (*n* = 16)	Group B (*n* = 16)	*P* value
Corrective carrying angle (°)	26.1 ± 6.4	23.5 ± 5.5	0.0011
Operation time, min	26.9 ± 5.1	27.5 ± 5.3	0.7017
Length of incision, cm	5.7 ± 0.6	5.3 ± 0.4	0.0744
Bellemore criteria			0.0458
Excellent	15 (93.7)	9 (56.3)	
Good	1 (6.3)	5 (31.2)	
Poor	0 (0)	2 (12.5)	

## Discussion

Due to the malunion of supracondylar humeral fractures, the most common late complication is cubitus varus deformity. Corrective operations are necessary because severe cubitus varus represents an unacceptable deformity and does not improve with remodeling (14–16). Various options for correction of distal humerus deformities, such as closing wedge osteotomy, dome osteotomy, medial opening wedge osteotomy, step-cut osteotomy, and reverse V osteotomy have been reported (17–19). However, lateral closing wedge osteotomy has been widely accepted by most orthopedic surgeons.

Although the degree of correction is properly performed, various fixation techniques will result in different corrective outcomes because the correction obtained can be compromised by inadequate fixation (20). Methods of fixation, such as K-wires, external fixation, screws or locking plates have also been published (21–24). Although the technique of K-wires may be faster and less invasive, a higher rate of return to surgery for loss of reduction was sometimes reported (3). Meanwhile, higher rates of infection and iatrogenic nerve injury are the disadvantage of K-wires method (25–27). The lateral external fixation could provide more stability to internal rotation in distal humerus without a second surgery for removal, which could also make a 3D correction for cubitus varus (28, 29). Compared with other fixation methods, the locking plate can rigidly stabilize the distal humerus, which is helpful to functional and esthetic outcome. In fact, to prevents prominence of the lateral condyle, the locking plate requires adequate medial translation and continuous lateral cortices (30). To date, there have been no studies directly comparing locking plate against K-wire and cast fixation technique.

In our present study, both locking plate and K-wires and cast fixation were effective and safe methods for the treatment of cubitus varus in children. There was no significant difference among operative duration and length of incision. Compared with K-wires and cast fixation method, better functional and cosmetic results can be achieved because the locking plate can rigidly stabilize the distal humerus. Even though K-wires and cast fixation can be thought a safer and easier method, a higher rate of infection was found in our K-wires and cast fixation group. Meanwhile, K-wires and cast fixation does not always result in stabilization of the osteotomy performed. Raney et al. reported a higher rate of return to surgery for loss of reduction with K-wires and cast fixation. The locking plate can reduce the risk being a kind of fixation that is completely internal. In group A, no neurovascular complication occurred and a statistically significant difference was noted based on the Bellemore criteria *(P = 0.0458).* Meanwhile, the postoperative carrying angle was significantly different at final follow-up between the 2 groups *(P < 0.01).* Moreover, age at surgery can influence the treatment outcome of cubitus varus in children. Javier et al. reported the results that Kirschner-wire vs. mini external fixator in lateral closing-wedge osteotomy for cubitus varus deformity and the patients were significantly younger in the Kirschner-wire group (31). In our present study, we found that we were willing to choose k-wires fixation for younger age patients, especially for under the age of 10.

The limitation of this study includes its retrospective nature and the small number of cases, thus prospective randomized controlled trials are needed in the next step. Besides, the follow-up period is short because the patients in our study were still at the stage of skeletal immaturity. Our preoperative planning for lateral closing-wedge osteotomy was based on radiographs, not computed tomographic or magnetic resonance imaging scans. Lastly, it is very significant that age at surgery can influence the outcome but we did not pay attention to age-related decision on the type of device to use.

## Conclusion

Both locking plate and K-wires and cast fixation after corrective lateral closing-wedge osteotomy are effective and safe methods for the treatment of cubitus varus in children. Compared with K-wires and cast fixation, we recommend the wedge osteotomy with lateral locking plate to treat the cubitus varus deformity because locking plate could achieve better functional and cosmetic results and stabilize the distal humerus rigidly.

## Data Availability

The original contributions presented in the study are included in the article/Supplementary Material, further inquiries can be directed to the corresponding author.
